# 3D Maps of Mineral Composition and Hydroxyapatite Orientation in Fossil Bone Samples Obtained by X-ray Diffraction Computed Tomography

**DOI:** 10.1038/s41598-018-28269-1

**Published:** 2018-07-03

**Authors:** Fredrik K. Mürer, Sophie Sanchez, Michelle Álvarez-Murga, Marco Di Michiel, Franz Pfeiffer, Martin Bech, Dag W. Breiby

**Affiliations:** 1 Department of Physics, Norwegian University of Science and Technology, Høgskoleringen 5, 7491 Trondheim, Norway; 2 Science for Life Laboratory and Uppsala University, Department of Organismal Biology, Evolutionary Biology Centre, Norbyvägen 18 A, 75236 Uppsala, Sweden; 3 ESRF – The European Synchrotron, 71 Avenue des Martyrs, 38000 Grenoble, France; 4 Sorbonne Université – CR2P – MNHN, CNRS, UPMC, 57 rue Cuvier, CP38, F-75005 Paris, France; 5 Lehrstuhl für Biomedizinische Physik, Physik-Department & Institut für Medizintechnik, Technische Universität München, 85748 Garching, Germany; 6 Department of Diagnostic and Interventional Radiology, Klinikum rechts der Isar, Technical University of Munich, 81675 München, Germany; 7 Department of Medical Radiation Physics, Clinical Sciences, Lund University, 22185 Lund, Sweden; 8 Department of Microsystems, University of South-Eastern Norway, 3184 Borre, Norway

## Abstract

Whether hydroxyapatite (HA) orientation in fossilised bone samples can be non-destructively retrieved and used to determine the arrangement of the bone matrix and the location of muscle attachments (entheses), is a question of high relevance to palaeontology, as it facilitates a detailed understanding of the (micro-)anatomy of extinct species with no damage to the precious fossil specimens. Here, we report studies of two fossil bone samples, specifically the tibia of a 300-million-year-old tetrapod, *Discosauriscus austriacus*, and the humerus of a 370-million-year-old lobe-finned fish, *Eusthenopteron foordi*, using XRD-CT – a combination of X-ray diffraction (XRD) and computed tomography (CT). Reconstructed 3D images showing the spatial mineral distributions and the local orientation of HA were obtained. For *Discosauriscus austriacus*, details of the muscle attachments could be discerned. For *Eusthenopteron foordi*, the gross details of the preferred orientation of HA were deduced using three tomographic datasets obtained with orthogonally oriented rotation axes. For both samples, the HA in the bone matrix exhibited preferred orientation, with the unit cell *c*-axis of the HA crystallites tending to be parallel with the bone surface. In summary, we have demonstrated that XRD-CT combined with an intuitive reconstruction procedure is becoming a powerful tool for studying palaeontological samples.

## Introduction

X-ray computed tomography (CT) based on attenuation contrast and/or phase contrast is increasingly used in palaeontology, owing to its ability of non-destructively providing internal 3D images of opaque materials^1,2^. In rare cases, the muscles are preserved, allowing their structure to be directly investigated^3^. The usual situation is however that soft tissues are not preserved, and the musculature of extinct animals can only be retrieved from the geometry and composition of the bone where the muscles once were attached. In extant vertebrates, muscles attach to the bones via collagen fibres embedded in the bone matrix to form muscle attachments (entheses)^4,5^. Muscle collagen fibres can be distinguished from the collagen fibres of the bone matrix based on their orientation, as the muscle collagen fibres are embedded in the bone matrix with an angle ranging from 0 to 60 degrees with respect to the bone surface normal^6,7^. Collagen fibres are positively birefringent structures and their orientation can be revealed using polarised light^8^. As muscle fibres are progressively embedded in the bone matrix during the animal development, they also mineralize^9^. The associated hydroxyapatite (HA) crystallites are negatively birefringent, and their orientation can also be revealed using polarised light in both extant and extinct physically sectioned specimens^10^.

Studies done by transmission electron microscopy (TEM) of bone from extant animals have shown that the HA crystallites are platelet-shaped and arranged in parallel layers aligned along the collagen fibre axis^11^. Research indicates that the diagenesis, i.e. the process in which biological materials degrade during fossilisation, alters the morphology of HA in such a way that needle-shaped crystallites in addition to platelet-shaped crystallites can be found^11^. However, there is strong evidence that the orientation of the HA crystallites is preserved during fossilisation^12,13^, which opens for investigating the bone microstructure and associated soft tissues, such as muscles in fossils^6,7^.

Until recently, reconstructions of fossil musculature have been based mainly on the interpretation of muscle scars at the surface of fossil bones^14,15^. Polarised light micrography^6^ and (propagation-based) phase contrast micro-CT^7^ have been used to map muscle insertions on fossilised bone samples. However, they both  have important limitations. In order to extract the information of HA orientation using polarised microscopy, the sample needs to be sectioned into thin slices, and thus destroyed. Conventional CT has insufficient resolution to study the nanoscale mineral orientation. High-resolution micro- or nano-CT, even if it may reach the appropriate resolution for studying crystallite shapes, has a limited field of view of about 1 mm at high resolution, which is insufficient for getting an overview of the muscle attachments in vertebrate fossil bones^16^. New approaches are therefore desired to extract the 3D orientation of the HA without damaging the fossils.

Bragg peaks seen in X-ray diffraction (XRD) contain information about atomic-scale crystal structures including the orientation of the crystal lattice^17^. XRD allows information to be gathered about the presence and concentration of different materials, as well as their morphology on the micro and nanoscale. These facts have made XRD the undisputed technique for resolving crystal structures, and for determining how materials respond to various external stimuli under *in-situ* conditions^17^. XRD is non-destructive, where other relevant techniques like transmission electron microscopy (TEM) or scanning electron microscopy (SEM) require destructive sample sectioning^12,18^. Fossil samples have previously been studied with XRD, however limited to powder diffraction^19,20^, which does not allow spatially resolved information about the mineral concentration nor crystallite orientation to be obtained.

New X-ray sources, detectors, and optics, combined with increased computing power, have led to a range of new scattering and imaging methods allowing spatially resolved structural information to be obtained from macroscopic bulk samples using X-ray CT with unconventional contrast mechanisms. Examples of these methods are small-angle X-ray scattering computed tomography (SAXS-CT)^21,22^, coherent diffractive imaging (CDI)^23–25^ and ptychography^26–28^. Recently, several approaches to spatially resolving material *orientations*, known as X-ray *vectorial imaging*^16,29,30^, have been developed, recently even with magnetic contrast^31^.

The combination of XRD and computed tomography (CT), often referred to as XRD-CT, denotes an emerging family of techniques where the information obtained from the diffracted radiation, rather than beam attenuation, is used to form contrast for 3D tomographic reconstructions^32^. XRD-CT is not yet commonly available for routine studies, but the technique is increasingly used in the materials sciences. The first publication using XRD-CT dates back to the late 1980s^32^, but because of the increased computational power and fast read-out low-noise detectors available today, more data can now be acquired and processed, which allows larger sample volumes to be measured and higher resolution images to be acquired. The fact that orientation information is contained in the scattering signal, is the salient feature that opens for 3D reconstructions of material *orientation*. Several publications employing the XRD-CT technique already exist, and frequently reported limitations include that the samples need to either consist of large crystallites compared to the resolved voxel size^33,34^, or a large number of isotropically oriented crystallites must be contained in each voxel volume^35^.

In this article, we report on the use of XRD-CT to gain insight into the microstructure of two fossil bones. We demonstrate for the first time that both the spatial distribution of minerals and the 3D orientation of HA can be obtained without damage to the fossils. Our XRD-CT approach opens new opportunities for non-destructive extraction of microstructural information from mineralized biological samples.

Samples were prepared from three bones: 1) The humerus of an extant tetrapod, *Desmognathus quadramaculatus*, for which the identification of a muscle insertion was known. The humerus was sectioned at the location of a muscle insertion^2,7^, and raster-scanning XRD mapping was performed on a 50 µm thin section to validate the concept of visualising changes of HA orientation at the site of a muscle insertion in a fresh bone. 2) The tibia of a fossil specimen, the 300-million-year-old *Discosauriscus austriacus*, where the location of a muscle insertion was known from observations using polarised light^30^. An XRD-CT measurement was made in the region of the muscle insertion (Fig. S1a), confirming that the diagenesis did not destroy the crystal structure of HA, which is crucial for our XRD-CT approach to work. 3) The humerus of a 370-million-year-old lobe-finned fish *Eusthenopteron foordi*^36^, which has remained un-sectioned but for which we know there are muscle insertions based on local high-resolution images produced by phase-contrast micro-CT^7^. The humerus of *Eusthenopteron foordi* has a complex shape and microanatomy^37^ which is representative of most fossil bones. To the best of our knowledge, this is the first time 3D XRD-CT has been used for non-destructive structural analysis on a complex-shaped fossil bone.

### XRD-CT reconstruction procedure

**Figure 1:**
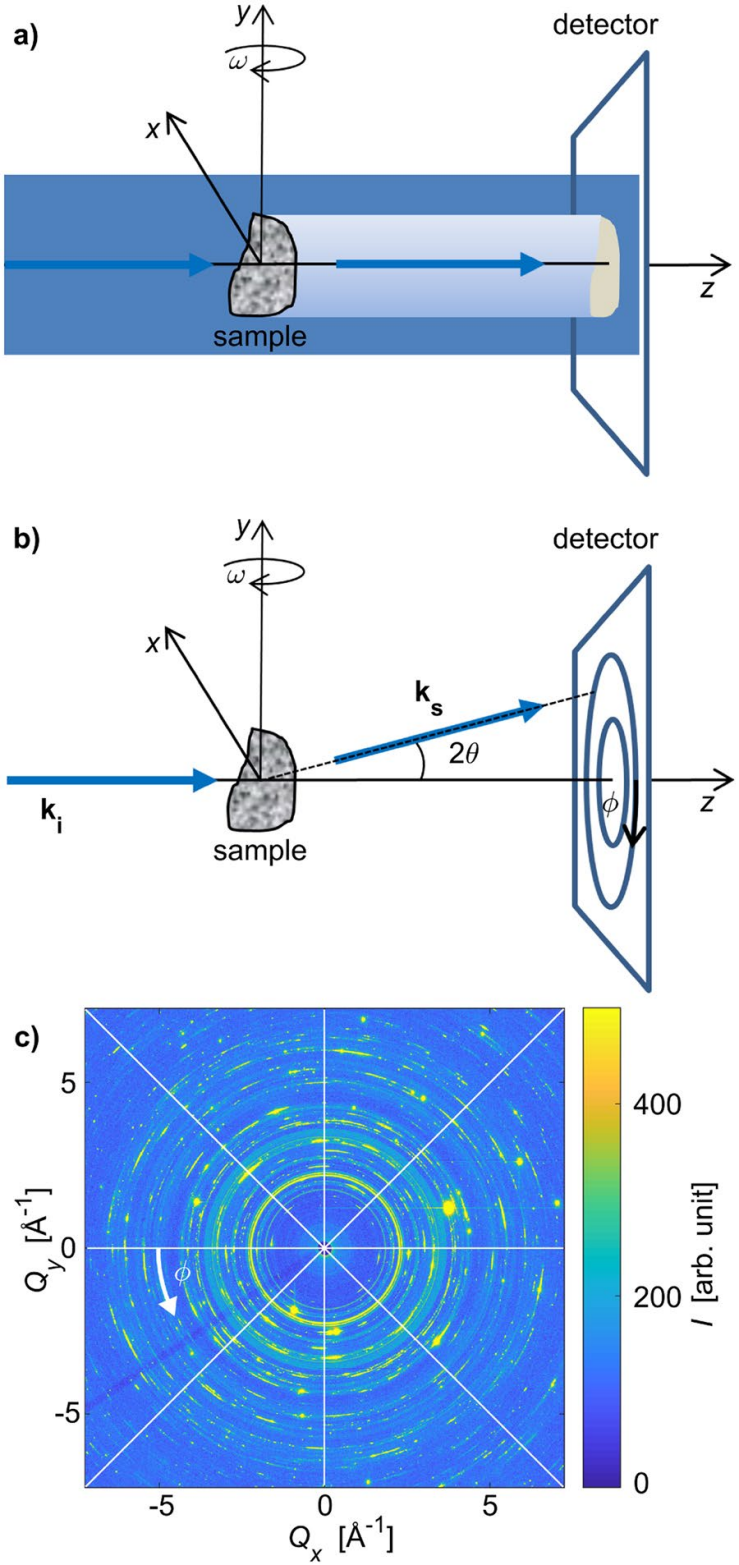
(**a**) Sketch of a parallel-beam attenuation-based CT setup with a rotating sample. A wide beam floods the sample, and the transmitted beam intensity is recorded on a detector. Through tomographic reconstruction the spatially resolved density of the sample is obtained. (**b**) Sketch of a generic XRD-CT setup as employed in our experiment. The collimated pencil beam from the synchrotron source with wavevector **k**_**i**_ enters the sample from the left. A fraction of the incoming beam is elastically scattered by the sample, and exits on the right as a scattered beam with wavevector **k**_**s**,_ while the remaining un-scattered photons exit the sample as a transmitted beam which is blocked by a beam-stop. Diffraction patterns were measured for different combinations of sample positions (*x, y*) and projection angles *ω*. (**c**) An example of a recorded diffraction pattern from the humerus of *Eusthenopteron foordi* (with linear intensity scale). Continuous Debye-Scherrer diffraction rings originating from HA can be seen, while the scattering from barite, calcite, quartz and pyrite is seen as bright spots. The white lines indicate sectioning of the diffraction patterns into azimuthal sections, used for tomographic reconstruction. 8 sectors are indicated for illustration purposes, while 64 sectors were used in the actual analysis. The beam stop support is seen as a dark line in the lower left region.

To understand the procedures we used for XRD-CT measurements and reconstruction, it is instructive to first reconsider the fundamentals of attenuation-CT, shown for parallel beam geometry in Fig. 1a. For a collimated monochromatic X-ray beam propagating in the *z*-direction, one can quantitatively describe the X-ray attenuation using Lambert-Beer’s law,

1 $${I}_{t}(x,\,y,\,\omega )={I}_{0}\exp (\,-\,\int \mu (x^{\prime} (\omega ),y^{\prime} ,z^{\prime} (\omega ))ds)$$

Here *I*_*t*_(*x, y, ω*) is the transmitted beam intensity, *I*_0_ the incoming beam intensity, *μ*(*x′, y′, z′*) the spatially resolved linear attenuation coefficient^38^. *x, y* and *z* are coordinates in the laboratory system (Fig. 1a), while *x*′, *y*′ and *z* refer to the internal sample coordinates. The sample coordinate system rotates during tomography with the sample projection angle *ω*, keeping *y′* = *y*. The integral is taken over the distance through the sample for a given ray. The transmitted intensity *I*_*t*_(*x, y, ω*) of the X-ray beam through the sample is measured for a wide range of projection angles *ω*, ideally covering 180°. The notation emphasizes that for each tomographic projection angle *ω*, the intensity is measured as a function of position (*x, y*). To reconstruct *μ*(*x′, y′, z′*) from the measured data, the fast and robust filtered back-projection (FBP) algorithm, which is based on the inverse Radon transform, is often used^38^.

In XRD-CT, the measured *diffracted* intensities, rather than the reduction in intensity (attenuation) of the direct beam, are used to reconstruct the scattering characteristics of each voxel in the sample volume (or cross-sectional area). Using carefully designed criteria (cf. SI), we extracted information from each 2D diffraction pattern using what we coin descriptors *I*_descriptor_ (*x, y, ω*). These descriptors are designed to return one single scalar value for each combination of (*x, y, ω*), which can then be fed into the FBP algorithm. Challenges arise because the scattering signal from each voxel volume in the sample changes with the projection angle. For retrieving the spatial distribution of minerals we define the descriptor ${I}_{\text{isotropic}}^{\text{hkl}}$ as the integrated intensity over all *ϕ* (Figs. 1b,c and S2) for a given diffraction lattice plane family *{hkl}* (i.e. a given scattering vector length *Q*). For the minerals being present in the bone samples with only few and large crystallites, resulting in tomograms with streak artefacts when reconstructing with the FBP algorithm, it proved useful to add several Bragg peaks for the composition analysis. Conversely, because the HA crystallites turned out to be small, numerous, and with wide orientation distributions, the descriptor for determining the spatial distribution of HA could be based on any of the HA Bragg peaks. The 002_HA_ reflection was selected for texture analysis because it has an easy interpretation as the unique axis of the hexagonal unit cell. We further define the descriptor *I*_total_ as the integrated intensity over all *ϕ* and *Q*, which is sensitive to the presence of any scattering compound in the sample.

The directional information contained in a diffraction pattern can be used to infer the orientation of the scattering mineral crystals. Two descriptors were designed to estimate the HA crystallite orientation, based on measurements with one or three tomography axes (Fig. S1). The single-axis approach was to first reconstruct the location of the HA crystallites based on the *meridional I*_||_ and *equatorial I*_⊥_ scattered intensities separately (cf. SI). Each descriptor yields its separate tomogram, showing the corresponding relative scattering of the HA. Because the HA was only weakly anisotropic, these tomograms were at first glance similar, but numerical comparisons indicated regions with a difference in the vertical and horizontal scattered signal. It proved useful to define an *orientational parameter κ* by

2 $$\kappa =\frac{{I}_{||}-{I}_{\perp }}{{I}_{||}+{I}_{\perp }}\,$$

to quantify orientation information. The other, multiple-axis, method of determining the HA crystallite orientation was to do several (here: three) full tomography scans with the sample mounted in different orientations with respect to the tomographic rotation axis. *I*_||_ from a given location (voxel) in the sample will remain essentially invariant during sample rotation, which is a requirement for tomographic reconstructions using FBP. Having measured the sample with three orthogonal orientations, giving three different tomograms each based on *I*_||_, gives the possibility of estimating an approximate *vectorial tomogram*, with one dominant HA crystallite orientation assigned to each of the voxels. For a more detailed explanation, cf. SI.

## Results

Results for the extant reference and the two fossil samples are presented. Both fossil samples, the tibia from *Discosauriscus austriacus* and the humerus from *Eusthenopteron foordi*, contained minerals of different crystallite sizes. The HA crystallites were small (compared to the reconstructed voxel size) and numerous, with many crystallites satisfying the diffraction condition simultaneously, regardless of sample position and orientation. The other minerals, secondarily present in the bone due to the fossilisation, had larger crystallites, giving rise to bright spots in the diffraction patterns (Fig. 1c). Consequently, we could derive composition maps for all minerals, while for HA, also orientational maps indicating the dominant orientation could be obtained.

### Scanning XRD of an extant salamander bone slice showing oriented HA crystallites

**Figure 2:**
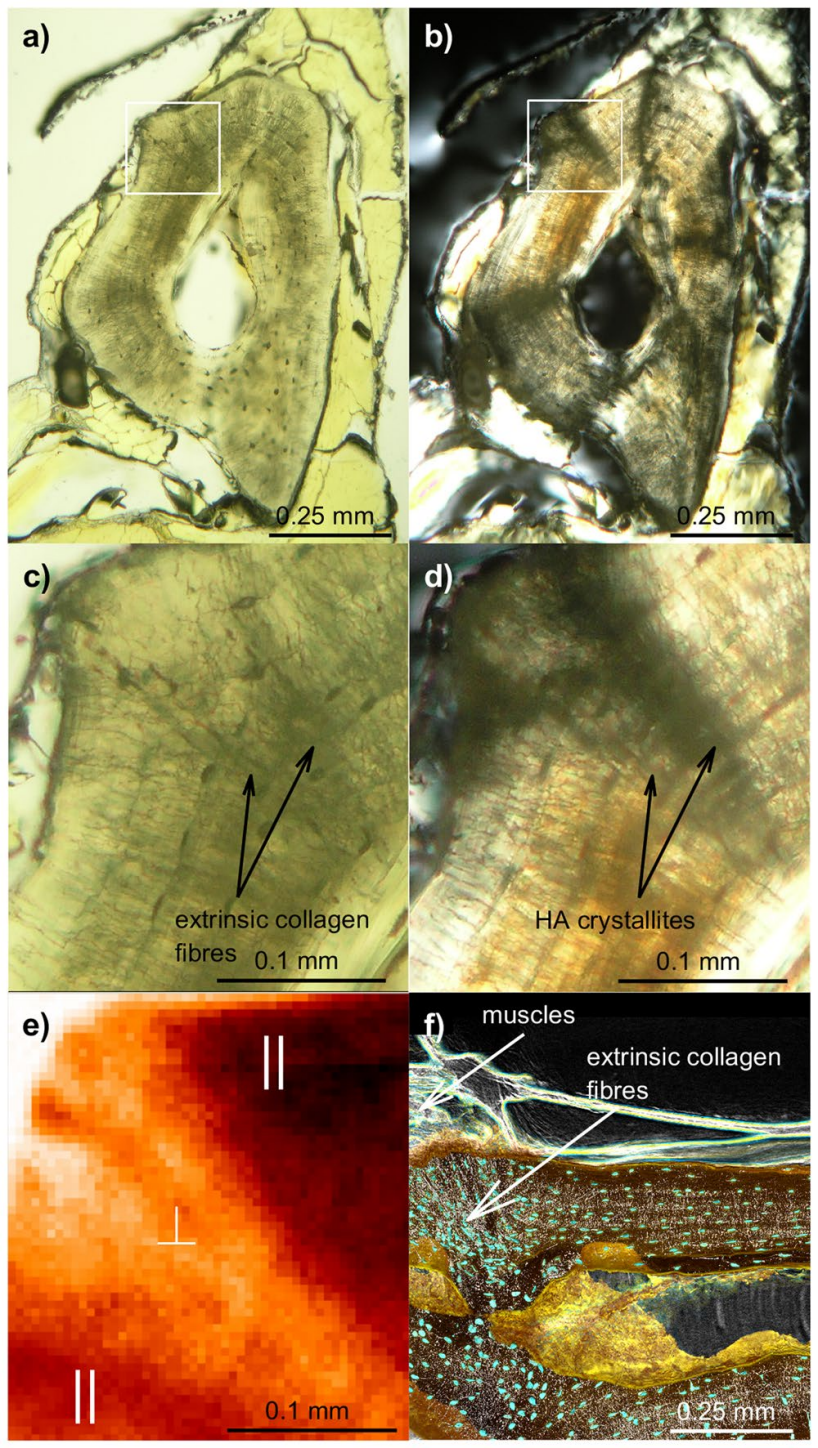
Humerus of the extant salamander *Desmognathus quadramaculatus*. (**a**,**b**) Natural and polarised light micrographs of a physically cut cross section through the sample, respectively, showing the region where the raster-scanning XRD map in (**e**) was made. (**c**,**d**) Details of the regions in (**a**) and (**b**), respectively. The arrows in (**c**) and (**d**) indicate locations of extrinsic collagen fibres and HA crystallites associated with muscle attachments. (**e**) Raster-scanning XRD map of the area indicated with a white rectangle in (**a**,**b**) and zoomed in (**c**,**d**), demonstrating that there is texture of the 002_HA_ reflection which can be mapped by XRD. Regions with either dominating meridional (out-of plane) diffraction or equatorial (in-plane) diffraction are marked by ∥ or $\perp$, respectively. In other words, brighter hues of red correspond to the HA *c*-axis being more inclined with respect to the long-axis of the bone. (**f**) Longitudinal phase-contrast CT section, clearly showing the presence of extrinsic fibres at the location where muscles attach to the bone surface (indicated by an arrow).

A 50 µm thin section made at midshaft in the humerus of the extant salamander *Desmognathus quadramaculatus* was studied to serve as a benchmark for the subsequent studies of fossil samples, demonstrating that the presence and orientation of HA in the vicinity of muscle attachments can be identified using XRD. Comparing Fig. 2a,b, it is apparent that polarised microscopy^8^ reveals a change of the collagen structure at the muscle attachments. The raster-scanning XRD measurement of the physically cut thin slice shown in Fig. 2e demonstrates that the orientation of the HA associated to the collagen fibres is indeed markedly different at the muscle attachment. Precisely, observing this structural modification in the diffracted signal suggests that XRD-CT can be used to study muscle attachments also in fossilised samples, which is the prime motivation for this study. Figure 2f shows the size and radial orientation of extrinsic fibres at the location of a muscle attachment, as studied with phase-contrast CT. It is important to note that even though phase contrast CT measurements resolve the bone structure to high detail, these images do not contain information about the HA orientation.

### 2D XRD-CT of the tibia of *Discosauriscus austriacus* showing oriented HA crystallites

**Figure 3:**
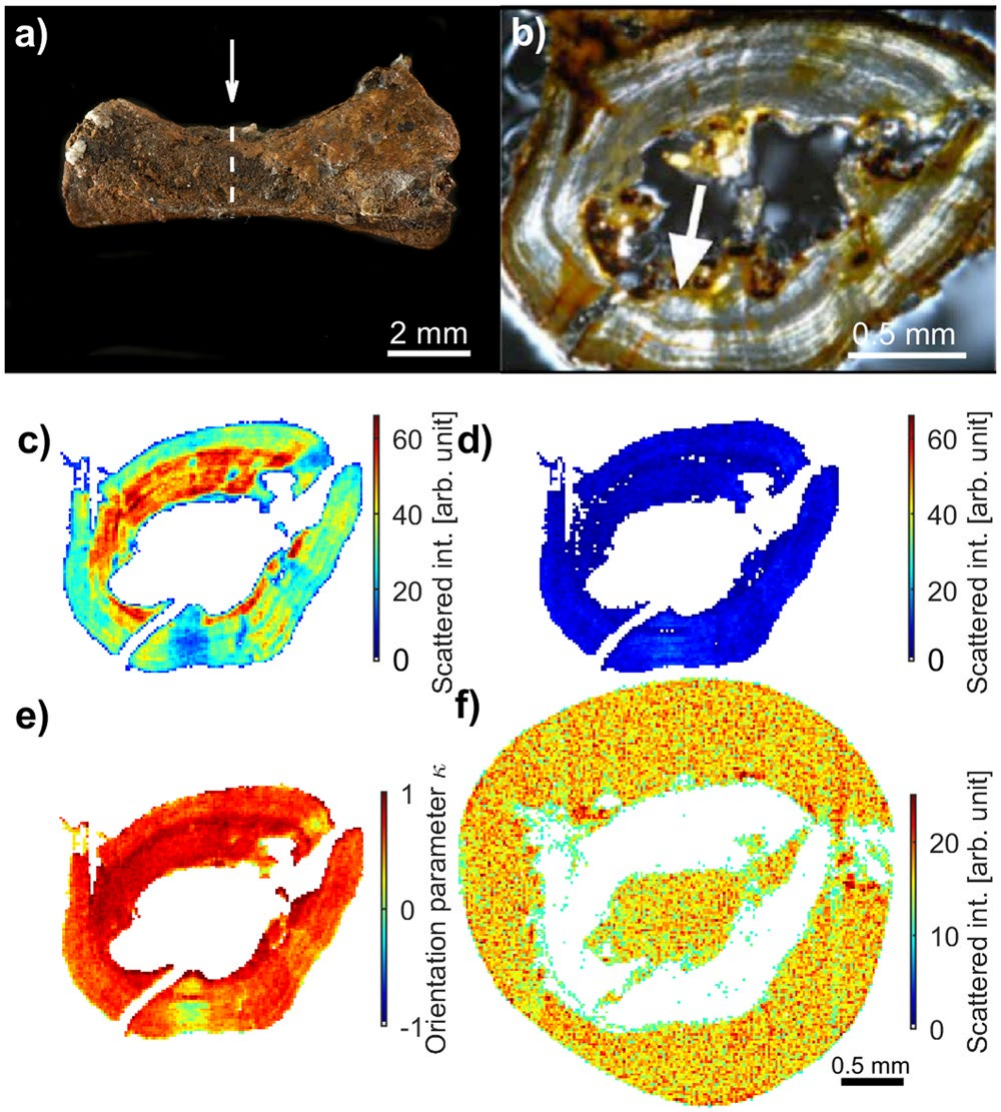
Tibia of the fossil tetrapod *Discosauriscus austriacus* (DE KO 58). (**a**) Photograph of the sample studied before sectioning. The arrow indicates the location of the cross-section measured with polarised light and XRD-CT. The photograph is provided by P. Loubry. (**b**) Polarised light micrograph of the physically-cut tibia cross section shown in (**a**). The arrow indicates the position of one of the muscle attachments, which corresponds to the region of maximum orientation contrast in the XRD-CT data, cf. (**e**). (**c**–**f**) *Vectorial* XRD-CT tomograms of the cross section in (**a**). (**c**) Tomograms based on ${I}_{||}^{002\text{HA}}$ and (**d**) ${I}_{\perp }^{002\text{HA}}$ (**e**) Normalized difference of the tomograms in (**a**) and (**b**), by using the orientation parameter *κ* (Equation 2). (**f**) Tomogram based on ${I}_{\text{isotropic}}^{\text{resin}}\,$for the amorphous resin signal.

The tibia of *Discosauriscus austriacus* (Fig. 3a) was chosen as a first fossil sample for observing muscle attachments because it has a simple long-bone shape. This gives a uniaxial distribution of the HA crystallites with the *c*-axis tending to be parallel to the long axis, except in the vicinity of muscle attachments. XRD-CT and images of thin sections, taken with polarized light, were compared on this fossil bone to check whether the diagenesis modifies the crystal structure of HA, which would alter the diffraction signal. A thin section was made at midshaft^39^ (Fig. 3a) where a muscle insertion could be visualised using polarised light (Fig. 3b). A 2D XRD-CT measurement (a single cross section) was obtained on the remaining half of the tibia embedded in polyester resin (Fig. S1). Bragg peaks corresponding to HA, as well as a broad scattering feature in *Q* corresponding to the surrounding amorphous resin, were indexed and used for tomographic reconstructions (Fig. 3c–f). HA in the bone and resin around the bone are distinctly visualized, illustrating how XRD-CT allows accurately mapping out regions containing different compounds.

The gross orientation of the HA crystallites in the tibia diaphysis of *Discosauriscus austriacus* was obtained by comparing tomograms reconstructed separately from the meridional (vertical) descriptor *I*_||_ and the equatorial (horizontal) descriptor *I*_⊥_. Note that albeit *I*_⊥_ does not fulfil the Bragg condition for all sample rotations (as the meridional descriptor *I*_||_ does), the horizontal scattering still clearly provided visible image features (Fig. 3d). As seen, the vertical scattering is more intense than the horizontal scattering, due to the preferred orientation of the HA crystallites. A map of the orientation parameter *κ* (Equation 2) indicates that the position of muscle insertions in the bone sample coincides with the region of minimum difference in *I*_⊥_ and *I*_||_ (Fig. 3e), as supported by a physically cut slice of the sample made in the same region viewed under polarised light (Fig. 3b).

### 3D XRD-CT of the humerus of *Eusthenopteron foordi* showing the location of minerals

**Figure 4:**
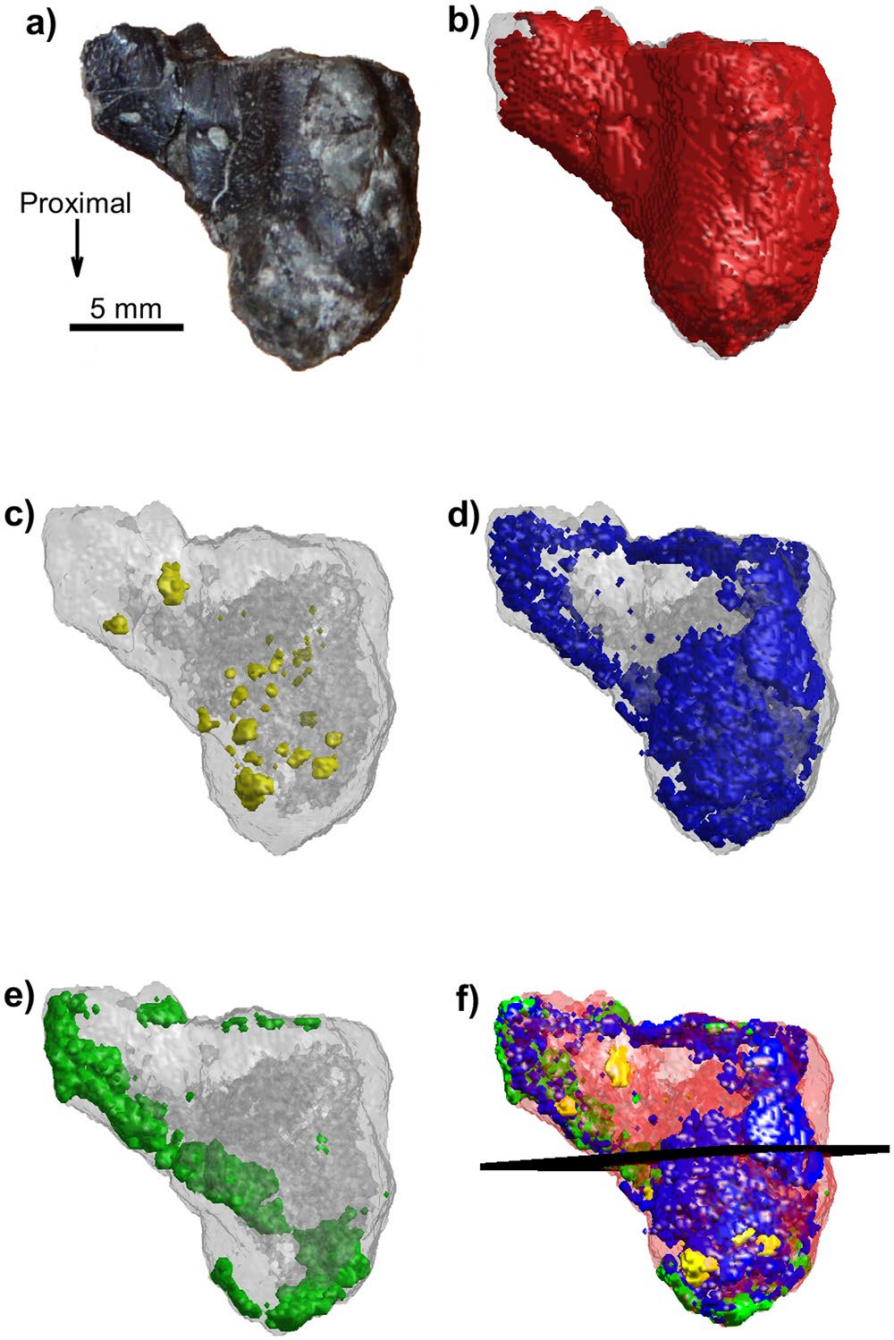
Humerus of the fossil lobe-finned fish *Eusthenopteron foordi* (NRM P246c) in mesial view. (**a**) Photograph of the sample studied. (**b**–**f**) 3D compositional tomograms of different minerals. (**b**) HA (red), (**c**) barite (yellow), (**d**) calcite (blue), (**e**) quartz (green). The grey shaded region in (**b**–**e**) corresponds to the total scattered signal *I*_total_ (integrated over the whole detector). Notice how the HA volume overlaps almost completely with the volume reconstructed by the total scattered signal. (**f**) Combined tomograms of all materials in (**b**–**e**), using the same colour coding, with HA semi-transparent for increased visibility. The plane plotted in the middle of the sample gives the location of the cross section shown in Fig. 5.

For the humerus of *Eusthenopteron foordi*, we performed three full XRD-CT measurements by laterally raster-scanning the whole sample for a large number of projection angles (cf. Methods). Bragg peaks corresponding to HA, barite, calcite, quartz and pyrite were indexed. Figure 4 shows a 3D mapping of the spatial mineral distribution in the humerus of *Eusthenopteron foordi*. Reconstructed tomograms based on *I*_isotropic_ for HA, barite, calcite and quartz are visualized separately, all with a surrounding semi-transparent region corresponding to a tomogram based on *I*_total_. The pyrite signal was too weak to give reliable tomograms. Note how the spatial distributions of the various minerals are complementary to each other, jointly filling the 3D region constituting the sample.

**Figure 5:**
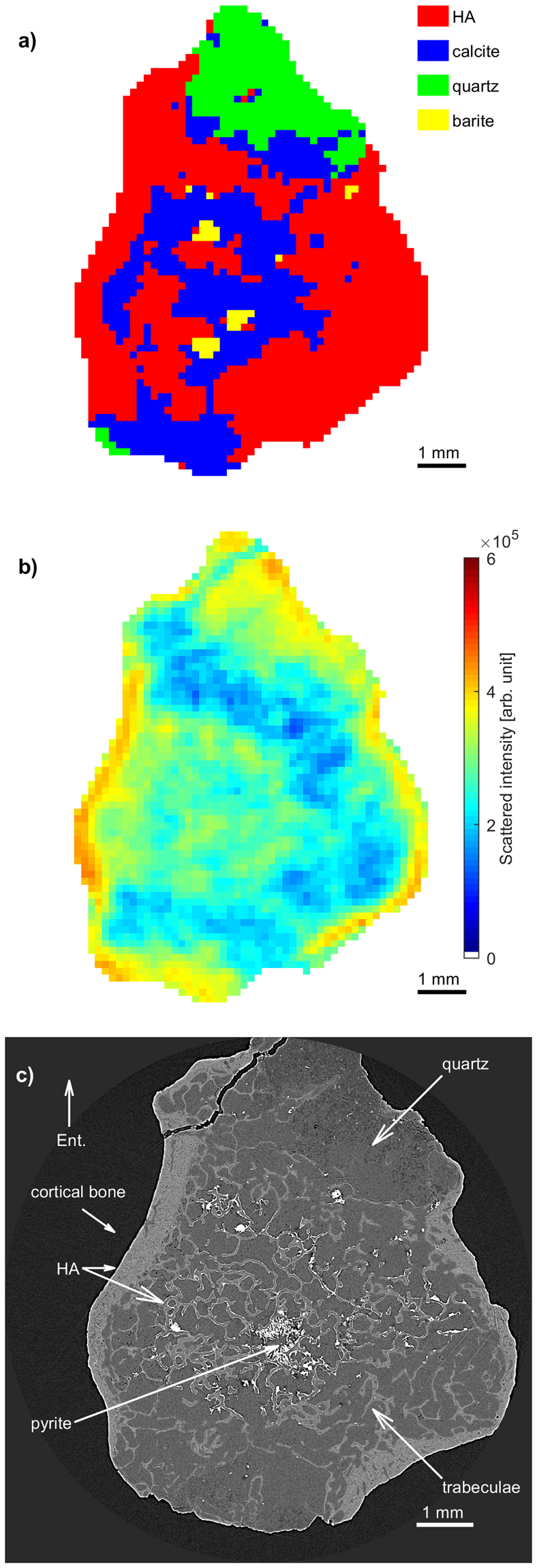
2D cross sections of *Eusthenopteron foordi* (NRM P246c) CT measurements, midshaft of the humerus, corresponding to the plane indicated in Fig. 4f. (**a**) XRD-CT cross section showing the dominating mineral present at each voxel. (**b**) XRD-CT cross section based on *I*_total_, being a rough estimate of the density of scattering material. (**c**) Phase contrast tomographic cross-section. Regions containing HA (cortical and trabecular bone), barite, quartz and pyrite have been labelled. Abbreviation: Ent., entepicondyle.

A cross-section of the reconstructed compositional XRD-CT tomograms (Fig. 4f) is shown in Fig. 5a. The total scattered signal *I*_total_ is shown in Fig. 5b. A phase-contrast tomogram is shown in Fig. 5c for comparison. Regions containing different minerals have been labelled, and albeit having much higher resolution, the locations of the different minerals in the sample are qualitatively consistent with what is found through the XRD-CT analysis. The HA crystallites are located in the cortical and cancellous bone, while the quartz covers large regions inside the rock matrix, in-between bony trabeculae. There are also small regions of barite (Fig. 4c), matching what is seen in the XRD-CT tomograms. The determination of the locations of pyrite could not be ascertained in the XRD-CT measurements, due to diffraction peak overlap.

### 3D orientation of HA in the humerus of *Eusthenopteron foordi* revealed by vectorial XRD-CT

**Figure 6:**
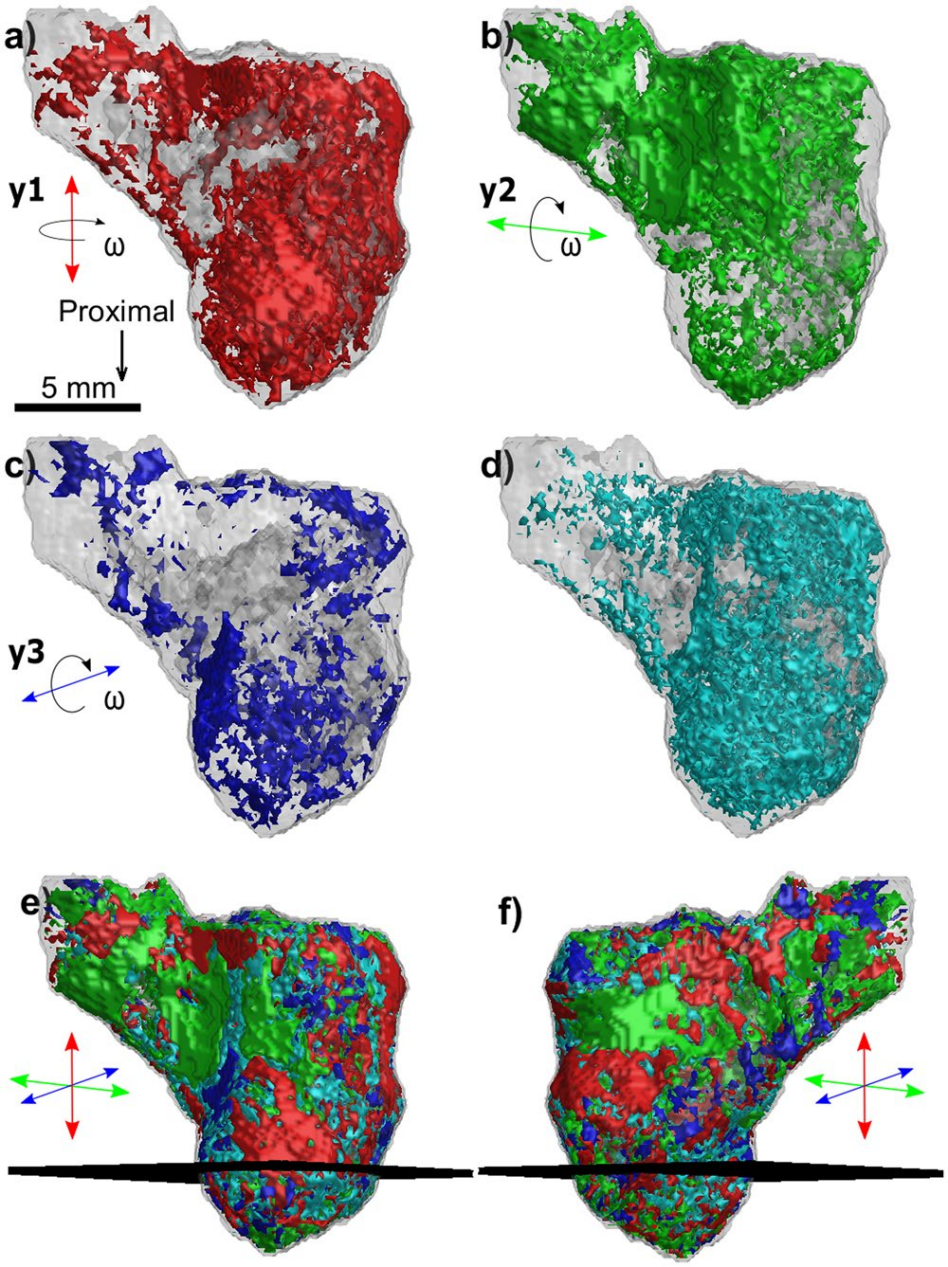
HA crystallite orientation in the bone of the humerus of *Eusthenopteron foordi* (NRM P246c) in mesial view. (**a**–**c**) Orientation-dependent 3D tomograms based on *I*_||_, indicating the locations of HA crystallites with the unit cell *c*-axis predominantly parallel to the experimental tomographic axis (indicated with an arrow for each case). To be judged significantly anisotropic, the intensity contribution along one axis had to be at least 30% higher than for the other two axes, otherwise the HA orientation in the voxel was considered to be isotropic. The dominating direction of the HA crystallite *c*-axis in different regions of the sample is color-coded and indicated by the arrows. Red indicates a proximal orientation of HA. Blue and green denote transverse orientations; blue follows the entepicondyle axis of the humerus while green shows an orientation orthogonal to the entepicondyle axis. (**d**) Tomogram highlighting voxels with isotropically oriented HA. (**e**,**f**) Preferred orientation of HA, visualized by a superposition of the three orthogonal tomograms, shown in two different views. The mesh plane at the lower half of the bone marks the position of the 2D section shown in Fig. 7.

**Figure 7:**
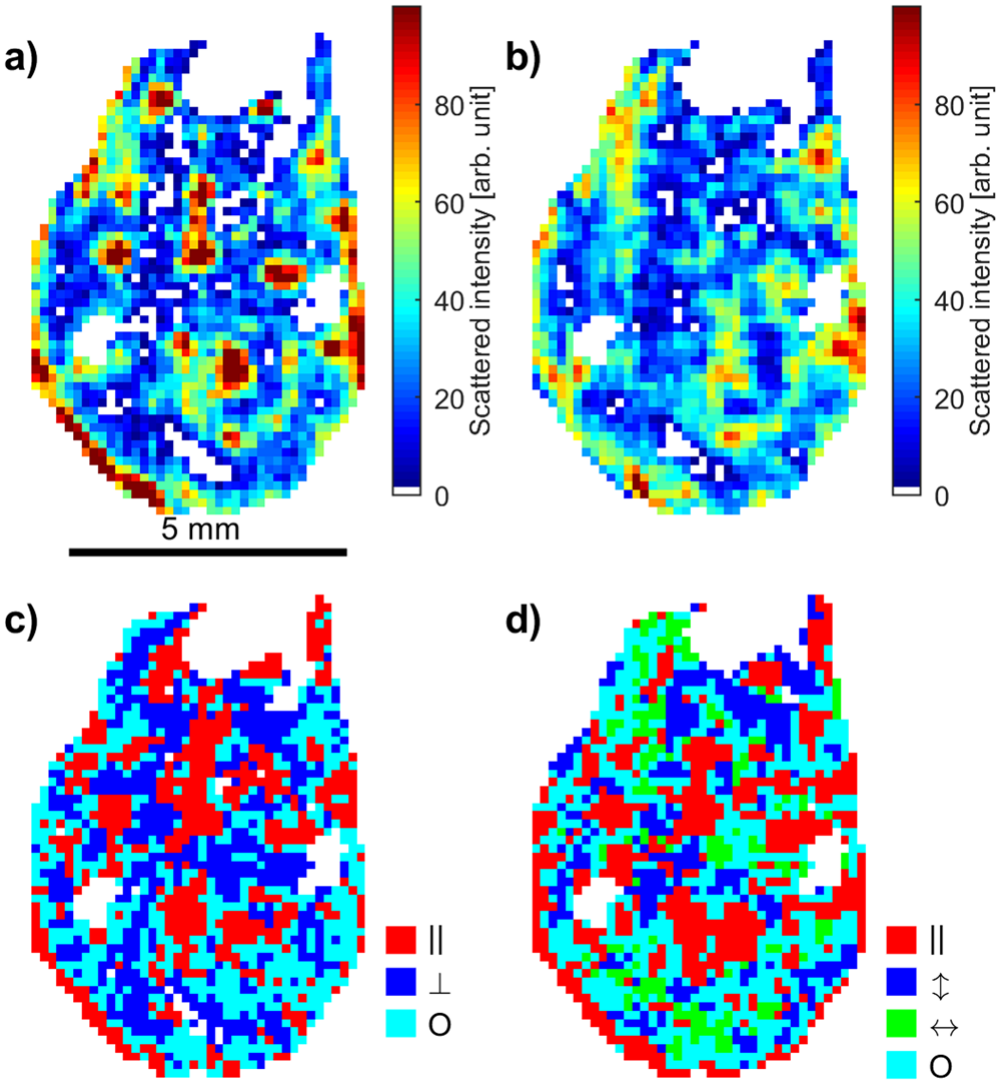
Cross section from 3D vectorial XRD-CT of the humerus of *Eusthenopteron foordi* (NRM P246c) yielding orientation information about the HA crystallites. (**a**) Single-axis tomogram based on ${I}_{||}^{002\text{HA}}$, (**b**) single-axis tomogram based on ${I}_{\perp }^{002\text{HA}}$, (**c**) single-axis orientation parameter *κ* (Equation 2). Red colour corresponds to dominating out-of-plane scattering, while blue (small regions) indicate dominating in-plane scattering. Cyan means that the in-plane and out-of-plane contributions are approximately equal. (**d**) Orientation information reconstructed from the multiple-axis tomogram using ${I}_{||}^{002\text{HA}}$ from each data set. This 2D section is a slice through the tomogram indicated by a plane in Fig. 6e,f. Results from both methods show consistency, and as expected, more details are visible in the multi-axis approach. Some empty regions in the sample (white) appear in all tomograms due to presence of other minerals than HA or low diffracted intensities ${I}_{||}^{002\text{HA}}$ or ${I}_{\perp }^{002\text{HA}}$.

With three XRD-CT datasets obtained at orthogonal orientations (axes labelled **y1**, **y2**, and **y3** in Fig. 6) for the humerus of *Eusthenopteron foordi*, the spatially resolved preferred orientation of HA could be estimated. The meridional descriptor ${I}_{||}^{002\text{HA}}$ for selecting intensity was applied to the 002_HA_ diffraction peak, giving for each of the three datasets an independent vectorial 3D tomogram emphasizing the regions having the highest density of HA crystallites oriented with the unit cell *c*-axis predominantly parallel to the actual tomographic axis. These tomograms are presented in Figs. 6 and 7, demonstrating that the *c*-axis of the HA unit cell tends to follow the external surface of the fossil bone, corroborating earlier reports^9^. As for the compositional tomograms, it gives credibility to the reconstruction algorithm that the tomograms independently show continuous regions that are complementary to each other.

A closer investigation of a 2D section of the humerus of *Eusthenopteron foordi* is given in Fig. 7, comparing orientation maps obtained using the single-axis XRD-CT method (with the tomographic axis perpendicular to the section, i.e. proximal) to the multiple-axis (three) orthogonal XRD-CT scans. The corresponding section is marked by a plane in Fig. 6e,f. Figure 7d shows the bone reconstructed from three datasets using *I*_||_ for the 002_HA_ reflection, i.e. a 2D version of the 3D figures in Fig. 6. As can be seen in Fig. 7d, the dominating HA orientation varies in the surrounding cortical bone, thereby reflecting both the orientation of the HA in the bone matrix and the HA associated to the extrinsic fibres of the muscle insertions. HA in the spongiosa in the middle of the 2D section seems to have a preferred orientation along the bone axis (proximal). For generating the orientation map in Fig. 7c, we used the orientation parameter *κ* (Equation 2), with the additional requirement that the intensity along one direction should be at least 30% higher in one direction than the others, or otherwise the scattering was considered isotropic. The results from the single- and multiple-axes approaches are seen to be consistent, with the latter approach of course giving additional in-plane information. A comparison of Fig. 7c,d exhibits a gratifying similarity of the regions consisting of HA crystallites oriented with the *c*-axis out of the 2D section plane. One limitation of using a single tomographic dataset is a reduced ability at distinguishing between dominating horizontal scattering, i.e. crystallite orientation in the 2D section plane, and isotropic scattering.

## Discussion

XRD-CT allows retrieving spatially resolved mineral identification and orientation information of semi-crystalline regions from non-destructive diffraction experiments. The diffraction contrast underlying XRD-CT thus provides information that cannot be obtained from conventional tomography. For the first time this method was applied, with success, to fossil hard tissues.

Using polarised light microscopy and scanning-XRD we demonstrated on a known muscle insertion in a physically cut thin slice of cortical bone from an extant salamander (Fig. 2) that the orientation of HA at muscle insertions could be mapped. We further showed that XRD-CT with a voxel size of 20 μm can be applied to fossil bones to map their muscle insertions. Indeed, a muscle insertion, previously identified using polarised light, could be observed in the tibia diaphysis of *Discosauriscus austriacus* by XRD-CT (Fig. 3). Significant equatorial scattering (*I*_⊥_) was observed, and tomograms of the diffracted signal showed explicitly that this scattering originated from regions that coincided with a muscle attachment. In other words, XRD-CT with a single tomography axis can therefore be able to locate the position of muscle attachments in fossil bones.

As XRD-CT for muscle attachment mapping seemed promising, we decided to apply this method to a long-bone of the 370 million-year-old lobe-finned fish *Eusthenopteron foordi*. The more complex microarchitecture of the humerus of *Eusthenopteron foordi* was revealed by combining three independent full XRD-CT measurements with orthogonal tomography rotation axes. Reconstructed tomograms based on diffraction contrast, using the FBP algorithm for different scalar “descriptors” (integrated intensities), demonstrated the identification and location of different minerals to a voxel size of 150 μm. Due to the comparably large size of the humerus of *Eusthenopteron foordi*, the measurement in 3D had to be done with a lower resolution to keep the measurement time manageable. Finding the locations of muscle attachments unfortunately proved to be more complicated at this resolution. Indeed, the orientation analysis revealed that the cortical bone of the humerus seems to contain larger regions with dominating scattering in either of the perpendicular directions. Smaller regions corresponding to muscle attachments, previously identified with phase contrast CT^7^, could not be observed conclusively in these XRD-CT 3D tomograms. Consistently with previous studies^40^, this implies that the preferred direction of the HA crystallite *c*-axis varies rapidly as function of position, thus requiring higher resolution and making the precise determination of muscle attachments much more difficult in 3D.

Using exclusively the meridional part of the 002_HA_ Debye-Scherrer diffraction ring, we have demonstrated that spatially resolved information on the preferred orientation of HA in both the cortical and trabecular bone can be retrieved. In the cortical bone of the humerus of *Eusthenopteron foordi*, the HA crystallites in the diaphysis did not seem to exhibit a global preferential orientation. Locally, the crystallites were either longitudinally oriented, transversally oriented or organized with no preferential orientation. This observation is certainly due to the non-tubular and more complex shape of the *Eusthenopteron foordi* sample as compared to the long-bone of *Discosauriscus austriacus*, and would require a more sophisticated measurement scheme to be fully resolved.

The analysis of fossil bone matrix ultrastructure allows interpretations on the paleobiology of animals, including bone growth rates^10,39^ and/or adaptations to biomechanical loads^41^. Indeed, because the bone surface of the tibia of *Discosauriscus austriacus* was smooth and the bone long axis was oriented parallel to the tomography axis, the 002_HA_ Bragg peak could mainly be observed in the meridional direction (*I*_||_), thereby revealing that the gross orientation of HA was essentially parallel to the tomography axis (Fig. 3), i.e. with the HA *c*-axis parallel to the long axis of the bone. This confirms the identification of a parallel-fibred bone interpreted as the result of a relatively slow bone deposition rate^39^. Of course, using multiple-axes full tomography measurements with the sample in different orientations with respect to the tomography axis would have enabled more precise evaluations of the spatially varying main crystallite orientation^16,29^ at the cost of a more complex and time-consuming experiment, which was not considered essential for the current purpose. We note that in addition to the biological information, XRD-CT also revealed details of the diagenesis. In the present study, we demonstrated that we could localize regions of calcite, barite and quartz resulting from the fossilisation.

Recently, other studies have been published^16,29,31^ on reconstructing 3D orientation from non-crystalline objects using extensive data collection and advanced reconstruction algorithms, based on a large number of tomographic axes giving huge datasets. However, in our case the favourable nature of the HA crystallites allowed a simpler data collection and reconstruction process, using only one or three tomography axes. Combining scalar “descriptors” with the commonly used FBP algorithm is intuitively simple and easy to implement. It facilitates the analysis if the crystallites of interest have a close to isotropic distribution, and if their size is small compared to the beam size (or equivalently, small compared to the reconstructed voxel size). As explained in detail, the minerals constituting the *Eusthenopteron foordi* sample were found to exhibit different orientation properties and crystallite sizes. Importantly, the HA crystallites were deduced to be small compared to the voxels measured, because their orientation distributions were smooth, even when diffraction patterns from small volumes near the sample edge were studied. The HA crystallites exhibited broad orientation distributions (Δ*ϕ* > 50°, cf. SI), and the direction of the preferred orientation relative to the tomography axes was found to vary smoothly and systematically throughout the sample. For our approach based on the FBP, the spatially slowly varying HA orientation was decisive for being able to carry out the vectorial tomographic reconstructions for this material. Two procedures for creating vectorial tomograms were considered, one method using three orthogonal tomographic axes, the other using a single axis. The equatorial scattering *I*_⊥_, being perpendicular to the sample rotation axis, varied strongly during sample rotation, and FBP reconstruction artefacts in the tomograms must be expected for this reason. Nevertheless, as demonstrated, convincing similarities were found between the vectorial tomograms reconstructed from single-axis and three-orthogonal-axes tomographic measurements. Future work could consider the possibility of adopting iterative tomography reconstruction methods, effectively fitting an entire computer-generated 3D model, including spatial orientation distributions, to fully exploit the information contained in the experimental diffraction patterns.

In the special case of the sample containing a high number of small weakly textured oriented crystallites, XRD-CT provides detailed composition tomograms down to the limiting parameters of this experiment, i.e. the beam diameter giving reconstruction voxels of size 150 μm. The detector pixel size of 400 μm × 400 μm caused a resolution in *Q* of about 0.015 Å^−1^. For the type of analysis performed, this rather low resolution in *Q* was an issue, as there were several overlapping Bragg peaks from the minerals. Of course, a higher detector resolution would demand even more from the data handling systems both during and after the experiment. A higher detector resolution would also allow extracting information about the crystallite size from the *width* of the Bragg peaks. Despite these limitations in this pioneering study, we have demonstrated for the first time that the spatially varying preferred orientation of HA in fossils can be obtained non-destructively.

The net exposure time for the XRD-CT experiments was approximately one hour for the tibia of *Discosauriscus austriacus* and approximately 24 hours for the humerus of *Eusthenopteron foordi*. There were additionally other time-consuming steps, e.g. calibration of the setup, alignment of samples before the start of each measurement and recording of dark current frames from the detector during the measurements. The total time spent on the experiments was therefore approximately 12 hours for the tibia of *Discosauriscus austriacus* and approximately 72 hours for the humerus of *Eusthenopteron foordi*. While the measurement strategy employed for these experiments was time consuming, we consider it likely that with the development of new high-brilliance synchrotron sources, more efficient detectors and read-out methods, XRD-CT in the near future can be performed at least one order of magnitude faster.

The current study demonstrates that XRD-CT is on the verge of becoming a powerful method for mapping in 3D the mineral composition and orientation in fossil samples. We showed here, to the best of our knowledge for the first time, that the information of muscle insertions could be retrieved with certainty in extant and fossil tetrapod bones. The muscle attachments are of vital importance to deduce how the extinct animals were moving through advanced biomechanical analysis. For the established method of polarized microscopy, it certainly is a huge disadvantage that scarce fossils have to be destroyed to extract the desired data. While the resolution in this study was too low to resolve the muscle attachments in the humerus of *Eusthenopteron foordi* in 3D, this pilot study still demonstrates the feasibility of extracting such information, even for old fossil bones. This is a considerable contribution towards the development of diffraction-based CT techniques for reconstructing the rearrangement of soft tissues through major evolutionary events. Despite the efforts that are needed to carry out an XRD-CT experiment and its subsequent analysis, we believe that the unique information that can be extracted from such experiments will prove the technique crucial for studying palaeontological, biological and synthetic functional materials in future.

## Methods

The specimen of *Desmognathus quadramaculatus* was collected under ethical guidelines for research study by Bruce *et al*.^42^. The humerus was dissected, dried and embedded in a polyester resin. The bone was sectioned using an annular diamond-powder saw. The observations were made under polarized light through a Zeiss Axiovert 35 optical microscope. Pictures were taken with an Olympus Camedia C5060 camera fixed on the microscope at Sorbonne University (UPMC, France). The acid-prepared^43^. specimen of *Discosauriscus austriacus* (DE KO 58) is stored in the research collections of the Comenius University (Bratislava, Slovakia). The limb bone was embedded in a polyester resin to be sectioned, using the same method as for *Desmognathus quadramaculatus*. The mechanically prepared specimen of *Eusthenopteron foordi* (NRM P246c) was provided by Naturhistoriska Riksmuseet (Stockholm, Sweden).

All XRD-CT measurements on the two fossil samples were performed at beamline ID15A at ESRF, using a monochromatic beam with a wavelength 0.143 Å (86.6 keV). 2D diffraction patterns were recorded on a Perkin-Elmer XRD 1621 N ES Series detector with an effective pixel size of 400 μm × 400 μm. The exposure time for each recorded diffraction pattern was 70 ms for the tibia of *Discosauriscus austriacus* and 35 ms for the humerus of *Eusthenopteron foordi*. One highly resolved tomographic cross section was measured in the tibia of *Discosauriscus austriacus*, transversally through the diaphysis of the sample, resulting in a reconstructed pixel size of approximately 20 μm × 20 μm. For the humerus of *Eusthenopteron foordi*, the entire sample volume was imaged with three independent full sets of XRD-CT measurements. The latter were performed with three approximately orthogonal orientations of the sample on the sample stage, allowing information about the preferred orientation to be extracted. For each tomographic projection angle *ω*, 100 steps in *x* and *y* and 80 rotation angles were used, resulting in an effective voxel size of approximately 150 μm × 150 μm × 150 μm. For further details about the XRD-CT measurements and reconstruction procedure, cf. SI.

Transmission raster-scanning XRD measurements were performed at beamline ID27 at ESRF. An orientation map was obtained in the vicinity of a muscle insertion in a thin physically cut section of the humerus of the (extant) salamander *Desmognathus quadramaculatus*. For these measurements, a beam with wavelength 0.374 Å (33.2 keV) and a 20 × 20 µm cross section was used. Diffraction patterns were collected on a MAR345 image-plate detector with 158 × 158 µm pixel size and integrated using the XRDUA software^44^. The complete 2D data set consists of 61 × 51 diffraction patterns.

## Electronic supplementary material

Supplementary information

## References

[1] Cunningham JA, Rahman IA, Lautenschlager S, Rayfield EJ, Donoghue PCJ (2014). A virtual world of paleontology. Trends Ecol Evol.

[2] Sanchez S, Ahlberg PE, Trinajstic KM, Mirone A, Tafforeau P (2012). Three-dimensional synchrotron virtual paleohistology: a new insight into the world of fossil bone microstructures. Microsc Microanal.

[3] Trinajstic K (2013). Fossil musculature of the most primitive jawed vertebrates. Science.

[4] Benjamin M (2002). The skeletal attachment of tendons—tendon ‘entheses’. Comparative Biochemistry and Physiology Part A: Molecular & Integrative Physiology.

[5] Benjamin M (2006). Where tendons and ligaments meet bone: attachment sites (‘entheses’) in relation to exercise and/or mechanical load. Journal of anatomy.

[6] Hieronymus TL (2006). Quantitative microanatomy of jaw muscle attachment in extant diapsids. Journal of Morphology.

[7] Sanchez S (2013). 3D microstructural architecture of muscle attachments in extant and fossil vertebrates revealed by synchrotron microtomography. PLoS One.

[8] Bromage TG (2003). Circularly polarized light standards for investigations of collagen fiber orientation in bone. The Anatomical Record.

[9] Bacon GE, Bacon PJ, Griffiths RK (1980). Orientation of apatite crystals in relation to muscle attachment in the mandible. J Biomech.

[10] Francillon-Vieillot HBV (1990). Microstructure and mineralization of vertebrate skeletal tissues. Skeletal Biomineralization: Patterns, Processes and Evolutionary Trends.

[11] Dumont, M., Pyzalla, A., Kostka, A. & Borbély, A. In *Biology of the sauropod dinosaurs: Understanding the life of giants* 150–170 (Indiana University Press, 2011).

[12] Zocco TG, Schwartz HL (1994). Microstructural analysis of bone of the sauropod dinosaur Seismosaurus by transmission electron microscopy. Palaeontology.

[13] Dumont M, Borbely A, Kaysser-Pyzalla A, Sander PM (2014). Long bone cortices in a growth series of Apatosaurus sp.(Dinosauria: Diplodocidae): geometry, body mass, and crystallite orientation of giant animals. Biological journal of the Linnean Society.

[14] Klembara J, Bartík I (1999). The postcranial skeleton of Discosauriscus Kuhn, a seymouriamorph tetrapod from the Lower Permian of the Boskovice Furrow (Czech Republic). Earth and Environmental Science Transactions of The Royal Society of Edinburgh.

[15] Molnar JL, Diogo R, Hutchinson JR, Pierce SE (2018). Reconstructing pectoral appendicular muscle anatomy in fossil fish and tetrapods over the fins‐to‐limbs transition. Biol Rev.

[16] Liebi M (2015). Nanostructure surveys of macroscopic specimens by small-angle scattering tensor tomography. Nature.

[17] Als-Nielsen, J. & MacMorrow, D. *Elements of modern X-ray physics*. 187–191 (Wiley, 2011).

[18] Brandon, D. G. & Kaplan, W. D. *Microstructural characterization of materials*. 261–331 (Wiley, 1999).

[19] Dumont M, Kostka A, Sander PM, Borbely A, Kaysser-Pyzalla A (2011). Size and size distribution of apatite crystals in sauropod fossil bones. Palaeogeography, Palaeoclimatology, Palaeoecology.

[20] Jörg S (2015). Two-dimensional X-ray diffraction as a tool for the rapid, nondestructive detection of low calcite quantities in aragonitic corals. Geochemistry, Geophysics, Geosystems.

[21] Schroer C (2006). Mapping the local nanostructure inside a specimen by tomographic small-angle x-ray scattering. Applied physics letters.

[22] Feldkamp J (2009). Recent developments in tomographic small‐angle X‐ray scattering. physica status solidi (a).

[23] Skjønsfjell, E. T. B. *et al*. High-resolution coherent x-ray diffraction imaging of metal-coated polymer microspheres. *Journal of the Optical Society of America A* **35**, (2017). 10.1364/JOSAA.35.0000A7 29328079

[24] Miao JW, Charalambous P, Kirz J, Sayre D (1999). Extending the methodology of X-ray crystallography to allow imaging of micrometre-sized non-crystalline specimens. Nature.

[25] Chapman HN, Nugent KA (2010). Coherent lensless X-ray imaging. Nature Photonics.

[26] Rodenburg, J. M. *et al*. Hard-x-ray lensless imaging of extended objects. *Phys Rev Lett* **98** (2007). 10.1103/PhysRevLett.98.034801 17358687

[27] Thibault P (2008). High-resolution scanning x-ray diffraction microscopy. Science.

[28] Esmaeili M (2013). Ptychographic X-ray Tomography of Silk Fiber Hydration. Macromolecules.

[29] Schaff F (2015). Six-dimensional real and reciprocal space small-angle X-ray scattering tomography. Nature.

[30] Schaff, F. *et al*. Correlation of X-Ray Vector Radiography to Bone Micro-Architecture. *Sci Rep-Uk* **4** (2014). 10.1038/srep03695 PMC3892438 24424256

[31] Donnelly C (2017). Three-dimensional magnetization structures revealed with X-ray vector nanotomography. Nature.

[32] Harding G, Kosanetzky J, Neitzel U (1987). X-ray diffraction computed tomography. Medical physics.

[33] Alvarez-Murga M (2011). Microstructural mapping of C60 phase transformation into disordered graphite at high pressure, using X-ray diffraction microtomography. J Appl Crystallogr.

[34] Poulsen HF (2012). An introduction to three-dimensional X-ray diffraction microscopy. J Appl Crystallogr.

[35] Bleuet P (2008). Probing the structure of heterogeneous diluted materials by diffraction tomography. Nature materials.

[36] Clack JA (1997). Devonian tetrapod trackways and trackmakers; A review of the fossils and footprints. Palaeogeogr Palaeocl.

[37] Sanchez, S., Tafforeau, P. & Ahlberg, P. E. The humerus of Eusthenopteron: a puzzling organization presaging the establishment of tetrapod limb bone marrow. *P Roy Soc B-Biol Sci* **281** (2014). 10.1098/rspb.2014.0299 PMC3973280 24648231

[38] Kak, A. C. & Slaney, M. *Principles of computerized tomographic imaging*. 49–117 (IEEE Pr., 1988).

[39] Sanchez S, Klembara J, Castanet J, Steyer JS (2008). Salamander-like development in a seymouriamorph revealed by palaeohistology. Biol Lett.

[40] Nakano T, Ishimoto T, Jee-Wook L, Umakoshi Y (2008). Preferential orientation of biological apatite crystallite in original, regenerated and diseased cortical bones. Journal of the Ceramic Society of Japan.

[41] De Margerie E, Sanchez S, Cubo J, Castanet J (2005). Torsional resistance as a principal component of the structural design of long bones: comparative multivariate evidence in birds. The Anatomical Record.

[42] Bruce RC, Castanet J, Francillon-Vieillot H (2002). Skeletochronological analysis of variation in age structure, body size, and life history in three species of desmognathine salamanders. Herpetologica.

[43] Štamberg S (2003). Chemical preparation of vertebrates from the Lower Permian of the BoskoviceFurrow *Acta Musei reginaehradecensis*. Series A, Scientiae naturales.

[44] De Nolf W, Vanmeert F, Janssens K (2014). XRDUA: crystalline phase distribution maps by two-dimensional scanning and tomographic (micro) X-ray powder diffraction. J Appl Crystallogr.

