# Supplementary material for: 3D Maps of Mineral Composition and Hydroxyapatite Orientation in Fossil Bone Samples Obtained by X-ray Diffraction Computed Tomography
Source: Sci Rep. 2018 Jul 3;8:10052. doi: 10.1038/s41598-018-28269-1 (PMC6030225; doi:10.1038/s41598-018-28269-1)
Supplement: Supplementary file 1 — Supplementary information [file 41598_2018_28269_MOESM1_ESM.pdf]

Supplementary Information

3D Maps of Mineral Composition and Hydroxyapatite Orientation in Fossil Bone Samples Obtained by X-ray Diffraction Computed Tomography

Fredrik K. Mürer[a], Sophie Sanchez[b,c,d], Michelle Álvarez-Murga[c], Marco Di Michiel[c], Franz Pfeiffer[e,f], Martin Bech[g&], Dag W. Breiby[a,h*&]

a) Department of Physics, Norwegian University of Science and Technology, Høgskoleringen 5, 7491 Trondheim, Norway
b) Science for Life Laboratory and Uppsala University, Department of Organismal Biology, Evolutionary Biology Centre, Norbyvägen 18A, 75236 Uppsala, Sweden
c) ESRF – The European Synchrotron, 71 Avenue des Martyrs, 38000 Grenoble, France
d) Sorbonne Université – CR2P – MNHN, CNRS, UPMC, 57 rue Cuvier, CP38, F-75005, Paris, France
e) Lehrstuhl für Biomedizinische Physik, Physik-Department & Institut für Medizintechnik, Technische Universität München, 85748 Garching, Germany
f) Department of Diagnostic and Interventional Radiology, Klinikum rechts der Isar, Technical University of Munich, 81675 München, Germany
g) Department of Medical Radiation Physics, Clinical Sciences, Lund University, 22185 Lund, Sweden
h) Department of Microsystems, University of South-Eastern Norway, 3184 Borre, Norway

* Corresponding author: Dag W. Breiby (dag.breiby@ntnu.no)
[&] Equal contribution

S1. Experimental details

The XRD-CT measurements were carried out at European Synchrotron Radiation Facility (ESRF) at beamline ID15A, using a monochromatic beam of energy 86.6 keV, corresponding to a wavelength of λ = 0.143 Å. Compound refractive lenses and slits were used to focus the beam to a square spot (see below for dimensions) on the sample. A diode in the beam path upstream of the sample was used to correct for variations in the incoming photon flux. The sample was mounted on a stage allowing translations in the *x* and *y* directions along with rotation of the sample by ω about the tomographic *y*-axis. A beam stop was placed in front of the detector to prevent the intense direct beam from damaging the detector. The area detector was a Perkin-Elmer XRD 1621 N ES Series, operated in 2 × 2 binning mode to achieve a frame rate of 20 Hz, with 1024 × 1024 pixels and an effective pixel size of 400 μm × 400 μm. The time-varying dark current of the detector was accounted for by recording dark images. Slight misalignments in the setup were numerically corrected using the scattering signal from a CeO_2 powder reference. Corrections for polarization and Lorentz factor were found to be negligible at the small 2θ angles studied. Absorption corrections were not made, due to the qualitative nature of the analysis.

The sample of *Discosauriscus austriacus* (Fig. S1a) was measured with the long bone axis parallel to the tomography axis *y*. The beam was focused to a 20 μm × 20 μm spot. The sample was scanned in *x* in steps of Δx = 20 μm and rotated about the *y*-axis by the angle ω in angular steps of 0.90°. A total number of 250 different *x* and 200 different ω were used, where $\omega \in$ [0,

180º]. Only one value of y was measured, giving a cross section of the diaphysis (Fig. S1a). These parameters provided pixels of approximate size 20 μm × 20 μm in the reconstructed single-slice tomogram.

For the sample of *Eusthenopteron foordi*, three independent full sets of XRD-CT measurements were performed with approximately orthogonal orientations of the sample on the sample stage (Fig. S1b), allowing information about the preferred orientation to be extracted. For each of the three orthogonal 3D tomographic measurements, the same measurement procedure was used. The resolution was reduced by refocusing the beam to a 150 μm × 150 μm spot. The position in y (“sample height”) was scanned across the sample in 100 steps, separated in y by Δy = 150 μm. For each position y, the sample was scanned in x in steps of Δx = 150 μm and rotated about the y-axis by the angle ω in angular steps of 2.22º. A total number of 98 different x and 81 different ω were used, where $\omega \in$ [0, 180º]. This provided voxels of size 150 μm × 150 μm × 150 μm in the reconstructed 3D tomograms. The 3D image registration problem of aligning the three datasets was solved by fitting three translation and three Euler angle parameters.

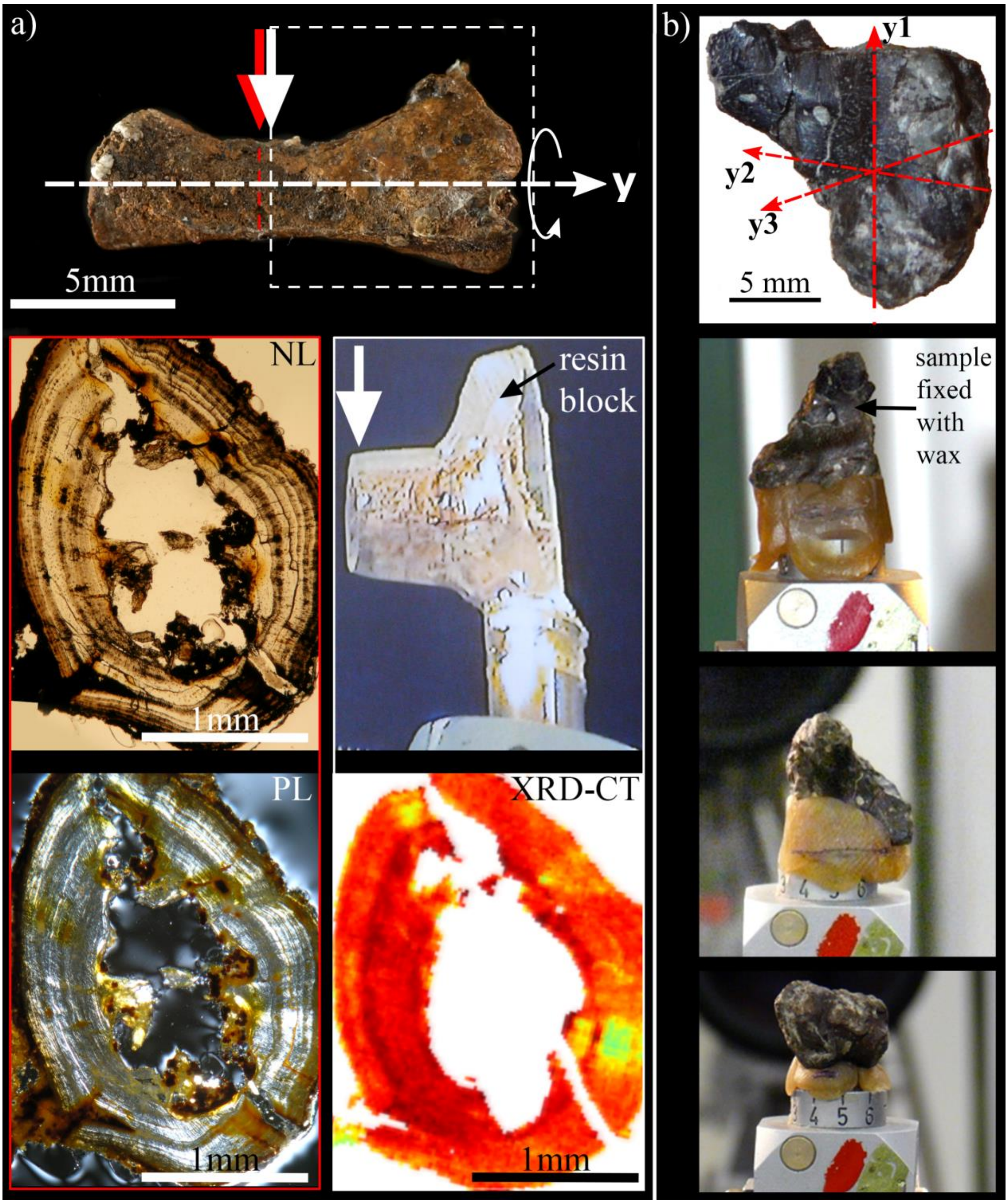

Figure S1 Fossil samples studied. a) Tibia of *Discosauriscus austriacus* (DE KO 58). The tomography axis used for XRD-CT measurement is indicated by the white arrow. The cross section measured with XRD-CT at midshaft is indicated by the red arrow. It is illustrated under natural light (NL) and polarised light (PL). The 2D virtual cross section measured with XRD-CT is indicated by the white arrow. It was made in close vicinity to the cross section in the shaft of the remaining half of the fossil embedded in a resin block. The photograph is provided by P. Loubry. b) Humerus of *Eusthenopteron foordi* (NRM P246c). The three approximately orthogonal tomography axes used are indicated by axes **y1**, **y2** and **y3**.

The 2D XRD-CT measurements of the tibia of *Discosauriscus* austriacus resulted in acquisition of approximately 100 GB of raw data, while the 3D XRD-CT measurements of the humerus of *Eusthenopteron foordi* resulted in approximately 3.6 TB of raw data. To reduce the amount of data, each diffraction pattern was divided into 64 sectors, and the intensity azimuthally averaged within each sector, thereby reducing the amount of data to 0.80 TB in total for both samples, at the cost of reducing the resolution in ϕ to $\Delta\phi = 360^o/64 \approx 5.6^o$. For efficient data processing, the intensity maximum of each Bragg peak was used to estimate the intensity contributions to the sinograms.

S2. A detailed description of XRD-CT

In an XRD-CT experiment, the observed intensity of the diffracted beam, rather than the reduction in intensity (attenuation) of the direct beam, is used to reconstruct the sample volume (or cross-sectional area). Whereas one usually uses a wide beam flooding the whole sample to record full projection images for attenuation CT, in the case of XRD-CT the sample is scanned using a narrow beam, and a 2D diffraction pattern is recorded for each position and orientation of the sample. A schematic setup of the XRD-CT experiment is shown in Fig. 1b, where far-field diffraction is recorded by a 2D-detector. The sample can be translated along the x and y axes as well as rotated about the y-axis with the sample rotation angle ω and illuminated by a monochromatic X-ray beam to measure diffraction from different parts of the sample for different directions.

The observed scattered X-ray intensity I in the far-field is proportional to the absolute square of the Fourier transform of the electron density in the sample[1], i.e., $I(\mathbf{Q}) \sim |F(\mathbf{Q})|^2$, where $F(\mathbf{Q})$ denotes the Fourier transform of the electron density. The scattering vector is defined as $\mathbf{Q} = \mathbf{k}_s - \mathbf{k}_i$, where $\mathbf{k}_i$ denotes the incoming and $\mathbf{k}_s$ the outgoing wave vectors. The magnitude of the scattering vector is given by $Q = |\mathbf{Q}| = 4\pi \sin(\theta)/\lambda$, where θ is half the total scattering angle 2θ and λ the photon wavelength. Because of the high energy of the radiation only scattering to small angles 2θ is observed. The reconstruction challenge is to assign - via tomographic methods - densities to the different voxels in the numerically reconstructed sample. Because diffraction patterns tend to exhibit circular features for polycrystalline samples, it is expedient to use polar coordinates, i.e., the measured intensity is a function of $I_{measured} = I_{measured}(\omega,Q,\phi,x,y)$ where ϕ is the azimuthal scattering angle, as illustrated in Fig. 1. Intensity distributions were extracted and tomograms obtained as described in detail (vide infra), using the MATLAB® built-in *inverse radon* function based on the filtered back projection (FBP) with a Ram-Lak filter.

S3. Data analysis

The approach explored in this study is to project each recorded diffraction pattern onto one single scalar value *("intensity descriptor")* $I_{\mathrm{descriptor}}(\omega,x,y)$, analogous to how one single transmitted intensity parameter $I_{\mathrm{t}}(\omega,x,y)$ is measured in conventional CT. A requirement is that the signal should be (at least approximately) invariant under rotations, as further discussed below. Then, we hypothesize that the computationally efficient and well-established filtered back-projection (FBP) reconstruction procedure can be inherited from attenuation CT. Clearly, there are many ways of reducing a diffraction pattern to a single scalar, as we shall elaborate in the following.

"Isotropic" descriptors
A particularly simple case is that of many isotropically oriented crystallites (a powder): The scattering can then be considered 1D ("powder-like"), and if choosing a particular $Q = Q_{\mathrm{hkl}}$ corresponding to a given Bragg peak, this diffracted intensity integrated over ϕ is a natural choice for the single scalar parameter. Mathematically, this can be expressed by

$$I_{\mathrm{isotropic}}^{\mathrm{hkl}}(\omega,x,y)=\iint \delta(Q-Q_{\mathrm{hkl}})I_{\mathrm{measured}}(\omega,Q,\phi,x,y)Qd\phi dQ \quad \text{(S1)}$$

For example, the 002_{HA} Bragg peak of an isotropically scattering region of HA can be integrated over all ϕ for each diffraction pattern, giving a reduced dataset $I^{002}{}_{\mathrm{isotropic}}(\omega,x,y)$, which can then be used to reconstruct in 3D where this crystalline phase is located inside the sample. This simplistic approach to diffraction contrast imaging is surprisingly powerful, and has even been used successfully in cases for a similar technique to XRD-CT, namely small-angle X-ray scattering computed tomography (SAXS-CT) where the scattering substance is *not* fully isotropic[2]. Note that for improved statistics, it is also in many cases possible to choose the summed intensity of several Bragg peaks, appropriately scaled for the unit cell structure factor, as the scalar parameter, a technique utilized in this work for reconstructing the tomograms of different minerals in the *Eusthenopteron foordi* sample (Fig. 4). The total scattered signal I_{total} is obtained by integrating I_{measured} over the whole detector, i.e.

$$I_{\mathrm{total}}(\omega,x,y)=\iint I_{\mathrm{measured}}(\omega,Q,\phi,x,y)Qd\phi dQ\,. \quad \text{(S2)}$$

In the more interesting case of preferentially oriented crystallites, the directional (vector) properties of **Q** must be accounted for. With an ensemble of crystallites exhibiting a shared preferred orientation, the diffraction pattern generally changes as the sample rotates. As mentioned, this is a complicating factor for tomography, while it also opens for vectorial imaging. If the sample contains only a few large single-crystalline grains, the algorithms described here are inadequate, as the diffraction signal will change too abruptly in ω and ϕ. We note that other groups have developed complementary methods for dealing with this special case[3]. However, if the crystallites are numerous, have a sufficiently low degree of preferred orientation, and small orientation gradients, it is possible to construct intensity descriptors that enable vectorial tomographic reconstructions based on filtered back-projection.

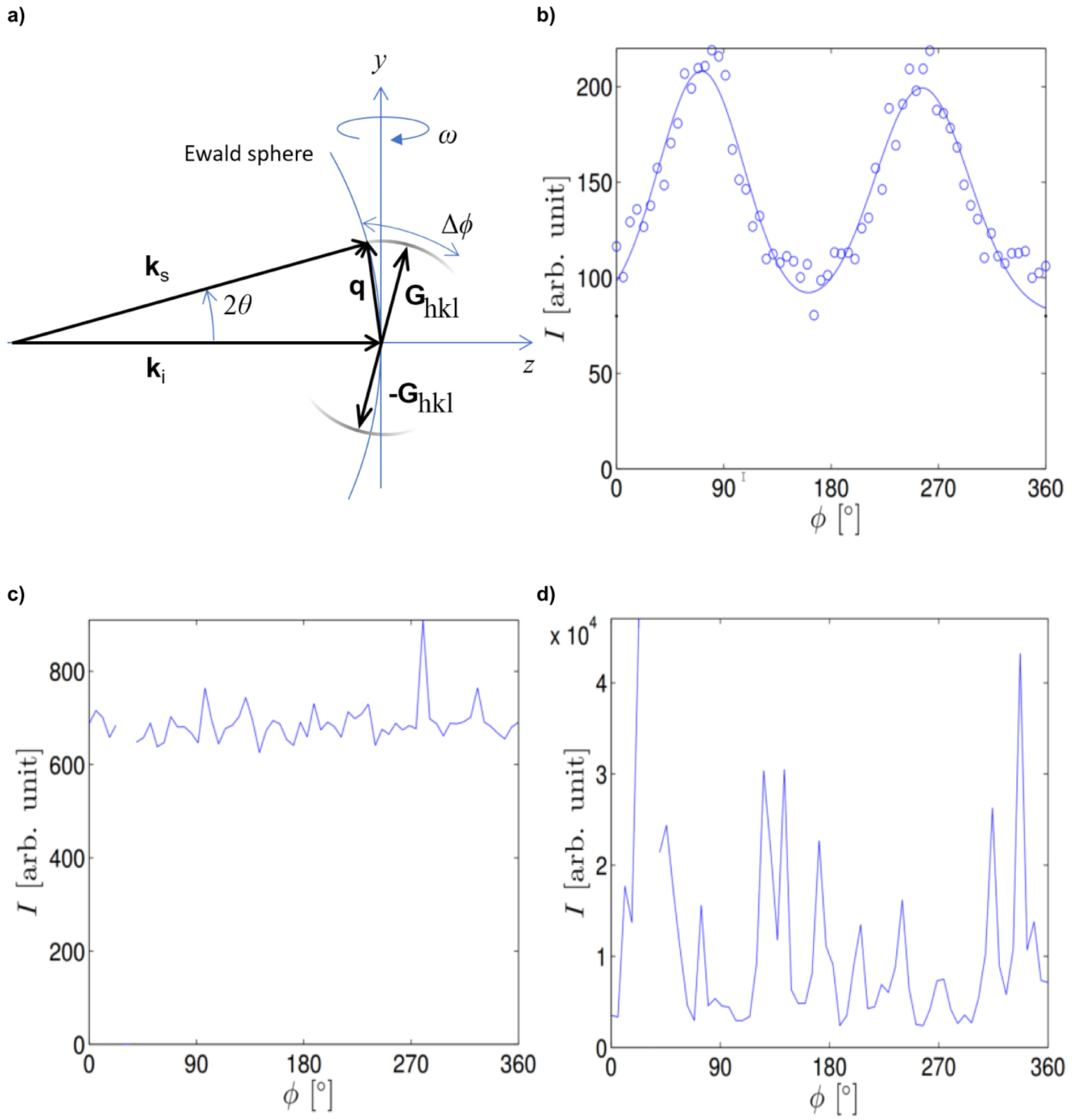

Figure S2 a) Illustration of the Ewald sphere for a polycrystalline sample with preferred orientation. The sketch illustrates the case of crystallites with a broad and continuous orientation distribution, such as the HA crystallites studied here. b) The 002_{HA} peak exhibits pronounced preferred orientation, with a peak width $\Delta\phi \sim 50^{\circ}$. c) Quartz Bragg peak. d) Calcite Bragg peak. The signals are qualitatively different: While the quartz 100_{qu} Bragg peak is essentially isotropic, the calcite 104_{ca} Bragg peak is also random, but fluctuates strongly as function of ϕ. The data thus suggests that there are many quartz crystallites, but only a few calcite crystallites. For both c) and d), the scattering at $\phi \sim 45^{\circ}$ is missing due to the beam stop support.

Meridional and equatorial descriptors

The idea coined "meridional descriptor" is essentially to integrate over a certain region of *Q*-space in such a way that the scattered intensity is minimally affected under sample rotation. Fig. S2a illustrates the Ewald sphere when considering diffraction from a single set of planes, with $\mathbf{G}_{hkl}$ and -$\mathbf{G}_{hkl}$ denoting a generic pair of reciprocal lattice vectors. In the general case when the sample is rotated, $\mathbf{G}_{hkl}$ will intersect the Ewald sphere for some sample rotation angles ω, causing diffraction peaks that can be observed on the detector. It is clear that Bragg peaks with preferred orientation in the *x*-*z* plane will change rapidly in intensity as the sample rotates. However, if $\mathbf{G}_{hkl}$ and -$\mathbf{G}_{hkl}$ point essentially in the meridional ("vertical"; **y**) direction, parallel to the tomography axis, the diffracted intensity will to a first approximation be independent of ω. Because the photon energy used is high, the Ewald sphere is almost flat, and the Bragg angles θ small. If θ is comparable or smaller than the width of the orientation distribution $\Delta\phi$, the vertical diffraction would be expected to be essentially constant when the sample is rotated, and thus we can define the meridional descriptor $I_{||}$ as

$$I_{||}^{hkl}(\omega, x, y) = \iint_{\phi \in \phi_{mer}} \delta(Q - Q_{hkl}) I_{measured}(\omega, Q, \phi, x, y) Q d\phi dQ \,. \quad \text{(S3)}$$

Formally, $I_{||}$ differs from $I_{isotropic}$ by integrating ϕ over only the meridional sectors of the detector. For example, for the 002_{HA} peak at θ = 1.2° for the energy used in this experiment, $I(\phi)$ has a width $\Delta\phi \approx 50°$, which is much larger than the corresponding Bragg angle, implying that if $\mathbf{G}_{002}$ is vertical, it will give essentially the same scattered intensity on the detector regardless of the sample orientation ω. The meridional descriptor proved essential for retrieving the orientation-dependent tomograms. It should be noted that the method described in this article, using the meridional descriptor, only provides estimates of the vectorial tomograms. In order to obtain a vectorial tomogram in a mathematically correct way, it is necessary to measure all three components of a lattice vector $\mathbf{G}_{hkl}$ for each voxel. The meridional descriptor does not measure these three components, but returns a measure of the statistical distribution of crystallites orientation within each voxel. This further implies that measuring samples with three orthogonal orientations is not sufficient for true vectorial tomography, but can provide useful qualitative results.

Similarly, the equatorial descriptor $I_{\perp}$ is defined for scattering to the equatorial detector sectors as

$$I_{\perp}^{hkl}(\omega, x, y) = \iint_{\phi \in \phi_{eq}} \delta(Q - Q_{hkl}) I_{measured}(\omega, Q, \phi, x, y) Q d\phi dQ \quad \text{(S4)}$$

For crystallites with a preferred orientation, $I_{\perp}$ from a sample volume element will generally change during sample rotation, and is therefore in general not suited for tomographic reconstruction using the FBP algorithm. However, when assumed that the crystallite orientation distributions are wide, and that the chosen Bragg peak for reconstruction is aligned along the tomography axis, one can obtain information about crystallite orientation, as shown for the samples from *Discosauriscus austriacus* and *Eusthenopteron foordi*.

As shown in Fig. S2b-d, the different minerals constituting the sample were found to exhibit qualitatively different orientation distributions. Nevertheless, 3D compositional tomograms for all minerals could be generated with few artefacts by using the isotropic "powder" intensity descriptor (Equation S1). The 3D fossil bone composition is shown with 3D XRD-CT tomograms in Fig. 4. It is particularly satisfying to note how the different minerals constituting

the sample fill complementary spatial regions. Unlike the other compounds, which form rather connected regions extending to the sample surface, the barite crystallites were found to be distributed with isolated regions solely in the sample interior.

Minerals present

Diffraction patterns representative of large regions of the sample were obtained by summing the diffraction patterns for many different sample positions and orientations, cf. Fig. S3 for the azimuthally averaged $I(Q)$.

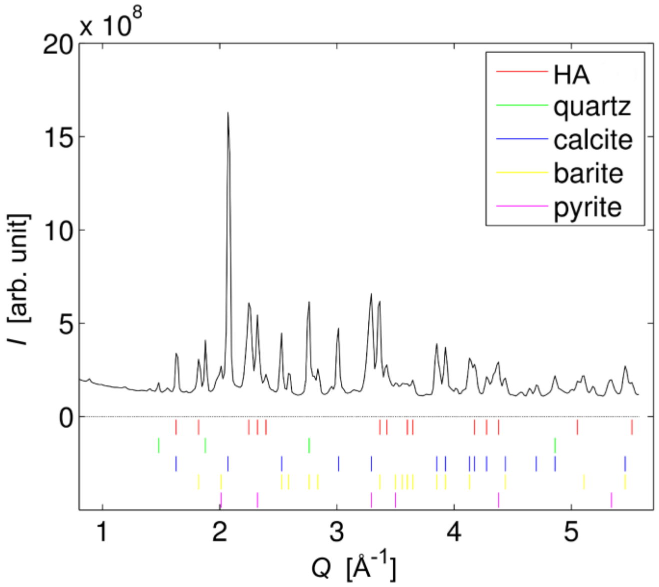

Figure S3 A 1D intensity global pattern for the humerus of *Eusthenopteron foordi* obtained by summing many individual azimuthally averaged diffraction patterns, used for identifying Bragg peaks suitable as signal for CT reconstruction. The most intense Bragg peaks belonging to the different minerals have been indicated with coloured lines. The background is seen to be essentially constant.

Bragg peak positions expected for the different minerals contained in the sample have been indicated with coloured lines in Fig. S3. The Bragg peak positions observed from the experimental data were compared with tabulated data[4-8], and all major Bragg peaks could be identified as originating from the listed five minerals. The relatively large pixel size of the detector combined with the small scattering angles caused a significant number of overlapping peaks, and only a few of the observed Bragg peaks could be extracted reliably and isolated for tomographic reconstruction, as listed in Table S1.

Table S1. FBP intensity descriptors and Bragg peaks used to reconstruct the XRD-CT tomograms. Overlapping peaks for similar *Q*-values are separated by “/”.

**Mineral**	**Intensity descriptor for FBP**	**Bragg peaks used for tomography**
hydroxyapatite (HA)	meridional / isotropic	002; 121/211; 202; 213/123
quartz	isotropic	011/101
calcite	isotropic	104; 202
barite	isotropic	311; 221/410
pyrite	not applicable	not applicable

The minerals in the sample, quartz, calcite barite and pyrite, were seen to exhibit qualitative differences in the scattering patterns in terms of preferred orientation, size and quantity. Large

crystallites necessarily cause streak artefacts in the XRD-CT tomograms, but it was possible to reduce these reconstruction artefacts by combining diffraction intensities from several Bragg peaks, as well as by combining the results from the three different datasets measured. Note that for *pyrite*, all except one observed Bragg peak were overlapping with peaks from HA and barite, which made it difficult to provide reliable pyrite tomograms. For the *HA* crystallites, the reconstructed tomograms were quantitatively similar regardless of which intensity descriptor that was used as input to the FBP. As shown with an example in Fig. S2b, the scattering signal from HA was smoothly varying with sample position and orientation, consistent with the presence of numerous crystallites, presumably of small size compared to the sample volume. A significant fraction of the crystallites was isotropically oriented, and using the isotropic descriptor resulted in tomograms with few streak artefacts.

For *quartz*, Fig. S2c shows that the crystallites diffract approximately equally to all ϕ for the 100_{qu} Bragg peak, suggesting that the quartz crystallites are isotropically oriented and that diffraction from a large number of crystallites is observed. The observed intensity from *calcite* fluctuates strongly with ϕ, as shown in Fig. S2d for the 202_{ca} Bragg peak. As for quartz, the calcite crystallites appear randomly oriented, but the Bragg intensities fluctuate strongly with ϕ, which indicates the presence of relatively few crystallites. The distribution of observed intensity from the *barite* 311_{ba} Bragg peak (not shown) exhibits sharp and strong peaks scattering randomly distributed in ϕ, also suggesting the presence of rather few crystallites.

References

1 Als-Nielsen, J. & MacMorrow, D. *Elements of modern X-ray physics*. 187-191 (Wiley, 2011).

2 Jensen, T. H. *et al.* Molecular X-ray computed tomography of myelin in a rat brain. *Neuroimage* **57**, 124-129, (2011).

3 Poulsen, H. F. An introduction to three-dimensional X-ray diffraction microscopy. *J Appl Crystallogr* **45**, 1084-1097, (2012).

4 Hughes, J. M., Cameron, M. & Crowley, K. D. Structural Variations in Natural F, Oh, and Cl Apatites. *Am Mineral* **74**, 870-876, (1989).

5 Levien, L., Prewitt, C. T. & Weidner, D. J. Structure and Elastic Properties of Quartz at Pressure. *Am Mineral* **65**, 920-930, (1980).

6 Graf, D. Crystallographic tables for the rhombohedral carbonates. *Am Mineral* **46**, 1283-1316, (1961).

7 Miyake, M., Minato, I., Morikawa, H. & Iwai, S. Crystal structures and sulphate force constants of barite, celestite, and anglesite. *Am Mineral* **63**, 506-510, (1978).

8 Bayliss, P. Crystal structure refinement of a weakly anisotropic pyrite. *Am Mineral* **62**, 1168-1172, (1977).
